# Identification of Protein–Protein Interactions via a Novel Matrix-Based Sequence Representation Model with Amino Acid Contact Information

**DOI:** 10.3390/ijms17101623

**Published:** 2016-09-24

**Authors:** Yijie Ding, Jijun Tang, Fei Guo

**Affiliations:** 1 School of Computer Science and Technology, Tianjin University, Tianjin 300350, China; wuxi_dyj@tju.edu.cn (Y.D.); tangjijun@tju.edu.cn or jtang@cse.sc.edu (J.T.); 2 Department of Computer Science and Engineering, University of South Carolina, Columbia, SC 29208, USA

**Keywords:** protein–protein interactions, protein sequence, feature extraction, amino acid contact, substitution matrix representation

## Abstract

Identification of protein–protein interactions (PPIs) is a difficult and important problem in biology. Since experimental methods for predicting PPIs are both expensive and time-consuming, many computational methods have been developed to predict PPIs and interaction networks, which can be used to complement experimental approaches. However, these methods have limitations to overcome. They need a large number of homology proteins or literature to be applied in their method. In this paper, we propose a novel matrix-based protein sequence representation approach to predict PPIs, using an ensemble learning method for classification. We construct the matrix of Amino Acid Contact (AAC), based on the statistical analysis of residue-pairing frequencies in a database of 6323 protein–protein complexes. We first represent the protein sequence as a Substitution Matrix Representation (SMR) matrix. Then, the feature vector is extracted by applying algorithms of Histogram of Oriented Gradient (HOG) and Singular Value Decomposition (SVD) on the SMR matrix. Finally, we feed the feature vector into a Random Forest (RF) for judging interaction pairs and non-interaction pairs. Our method is applied to several PPI datasets to evaluate its performance. On the S.cerevisiae dataset, our method achieves 94.83% accuracy and 92.40% sensitivity. Compared with existing methods, and the accuracy of our method is increased by 0.11 percentage points. On the H.pylori dataset, our method achieves 89.06% accuracy and 88.15% sensitivity, the accuracy of our method is increased by 0.76%. On the Human PPI dataset, our method achieves 97.60% accuracy and 96.37% sensitivity, and the accuracy of our method is increased by 1.30%. In addition, we test our method on a very important PPI network, and it achieves 92.71% accuracy. In the Wnt-related network, the accuracy of our method is increased by 16.67%. The source code and all datasets are available at https://figshare.com/s/580c11dce13e63cb9a53.

## 1. Introduction

Protein–protein interactions (PPIs) are fundamental importance to discover the molecular mechanism in biological systems. Identification of PPIs is important for elucidating protein functions and researching biological processes in a cell. In recent years, many prediction methods have been developed for the large-scale analysis of PPIs. Generally, these technologies refer to three categories of information, such as co-evolution information, natural language processing, and protein sequence feature.

Lots of methods analyze the co-evolution trend of protein–protein interactions [1,2,3,4,5,6,7,8]. They extract the evolution information of homologous proteins via multiple sequence alignment. It was possible for them to evaluate the relationship between protein pairs by linear correlation coefficient, the similarity measurement of phylogenetic trees or a log-likelihood score. Several technologies have been developed to find PPI evidence from PubMed abstracts, based on Natural Language Processing (NLP) [9,10]. According to a certain semantic model, it automatically extracts relevant pieces of information from literature, as a large number of known PPIs are stored in biology and medicine relevant scientific literature.

However, these methods of co-evolution are very difficult to compute because they need a large number of homology proteins. The problem of NLP is that PPI information can be missing from literature, thus prediction may be incomplete. A large number of studies accurately predict PPIs using protein sequence features to describe amino acids. Utilizing machine learning methods in this task, one of the most important computational challenges is to extract useful features from protein sequences. Guo et al. [11] use auto-correlation (AC) values of seven different physicochemical scales to describe an amino acid sequence. This method has been applied to predict the database of S.cerevisiae PPIs. Shen et al. [12] describe a protein sequence by amino acid groups, and its feature vector is formed by the occurrence of conjoint triads (CT). Zhou [13] and Yang [14] split the amino acid sequence into ten local regions of varying length and their compositions are represented by multiple overlapping continuous and discontinuous interaction information within one protein sequence. For each local region, they calculate three local descriptors (LD), such as composition (C), transition (T) and distribution (D). On the basis of LD, You et al. [15,16] expand the range of description by constructing multi-scale local descriptor (MLD) regions, and achieve higher prediction accuracy of the S.cerevisiae PPI dataset. Huang et al. [17] use BLOSUM62 [18] to construct a new matrix representation from the protein sequence, and achieve higher prediction accuracy on the Human PPI dataset. Existing approaches use physical and chemical properties of amino acids, position information of amino acids and evolutionary information to represent protein sequences. Wong et al. adopt the Physicochemical Property Response Matrix combined with the Local Phase Quantization descriptor (PR-LPQ) [19] as the feature of the protein sequence. However, they do not consider the contact information between various types of amino acids, which is important information to predict PPIs. Therefore, we will use amino acid contact information to improve the prediction accuracy on PPI identification.

In this paper, we propose a novel matrix-based protein sequence representation approach for predicting PPIs, using amino acid contact information to improve prediction accuracy and an ensemble learning method for classification. First, we construct the Amino Acid Contact (AAC) matrix, based on 6323 protein–protein complexes from a Protein Data Bank. We use the AAC matrix to represent the protein sequence as a Substitution Matrix Representation (SMR) matrix. Then, we extract the feature vector by applying Histogram of Oriented Gradient (HOG) and Singular Value Decomposition (SVD) algorithms on the SMR matrix. Finally, we feed the feature vector into Random Forest (RF) for judging interaction pairs and non-interaction pairs.

For the performance evaluation, our method is applied to the S.Tcerevisiae PPI dataset. The prediction results show that our method achieves 94.83% accuracy and 92.40% sensitivity. Compared with existing methods, the accuracy of our method is increased by 0.11 percentage points. Further demonstrating the effectiveness of our method, we also test it on the H.pylori PPI dataset. Our method achieves 89.06% accuracy and 88.15% sensitivity, the accuracy of our method is increased by 0.76%. On the Human PPIs dataset, our method achieves 97.60% accuracy and 96.37% sensitivity, and the accuracy of our method is increased by 1.30%. In addition, we test our method on an important PPI network, and it achieves 92.71% accuracy. In the Wnt-related network [12,20], accuracy of our method is increased by 16.67%, compared to the method of CT [12]. We also use the S.cerevisiae PPI dataset to construct a model to predict the other five independent species PPI datasets. Compared with the state-of-the-art works, the accuracy of our method is increased by 1.63% overall.

## 2. Results

In our experiment, we test our method on eight different PPI datasets to evaluate the performance of our proposed approach. Benchmark PPI datasets include one S.cerevisiae dataset, two H.pylori datasets, one Human dataset, one C.elegans dataset, one E.coli dataset, one H.sapiens dataset, and one M.musculus dataset. First, we independently analyze the performance of two protein representations, such as the Histogram of Oriented Gradient (HOG) and Singular Value Decomposition (SVD). Second, we compare our method with other outstanding methods in the S.cerevisiae, H.pylori and Human datasets. Then, we use S.cerevisiae PPIs dataset to construct a model to predict the other five independent species PPI datasets. Our proposed method achieves a high performance on S.cerevisiae, H.pylori and Human datasets, so we evaluate the prediction performance of our model on five independent testing datasets. Our experiments suggest that experimentally identified interactions in one organism are able to predict interactions in other organisms. In addition, we test our method on an important PPI network, and compare it to state-of-the-art works. We use primary experimental information to predict a real PPI network, which is assembled by pairwise PPI data. At last, we analyze the performance of different protein representation approaches by our method.

### 2.1. PPI Datasets

We test on eight different PPI datasets for evaluating the performance of our proposed approach.

The first PPI dataset, described by You et al. [16], is collected from the S.cerevisiae core subset in the database of interacting proteins (DIP) [21]. They remove the protein sequence, which is more than 40% sequence identity, to one another or fewer than 50 residues. The remaining 5594 pairs of proteins formed the final positive dataset. In addition, non-interacting pairs are selected uniformly based on an assumption that proteins occupying different subcellular localizations do not interact. Finally, the negative dataset is consisted of 5594 protein pairs, and their subcellular localization are different. The positive and negative datasets are combined into a total of 11,188 protein pairs.

The second PPI dataset, described by Martin et al. [22], is composed of 2916 H.pylori protein pairs (1458 interacting pairs and 1458 non-interacting pairs).

The third PPI dataset is collected from Human Protein References Database (HPRD) as described by Huang et al. [17]. Huang et al. constructed the Human dataset by 8161 protein pairs (3899 interacting pairs and 4262 non-interacting pairs).

The other five datasets include C.elegans (4013 interacting pairs), E.coli (6954 interacting pairs), H.sapiens (1412 interacting pairs), M.musculus (313 interacting pairs), and one additional H.pylori dataset (1420 interacting pairs) used by Zhou et al. [13]. These species-specific PPI datasets are employed in our experiment to verify the effectiveness of our proposed method.

### 2.2. Evaluation Measurements

To test the robustness of our method, we repeat the process of a random selection of training sets and test sets, model-building and model-evaluating. This process is fivefold cross validation. There are seven parameters: overall prediction accuracy (ACC), sensitivity (SN), specificity (Spec), positive predictive value (PPV), negative predictive value (NPV), weighted average of the PPV and sensitivity ($F_{score}$), Matthew’s correlation coefficient (MCC), which are defined as follows:

(1) $$ACC=\frac{TP+TN}{TP+FP+TN+FN},$$

(2) $$SN=\frac{TP}{TP+FN},$$

(3) $$Spec=\frac{TN}{TN+FP},$$

(4) $$PPV=\frac{TP}{TP+FP},$$

(5) $$NPV=\frac{TN}{TN+FN},$$

(6) $$F_{score}=2\times \frac{SN\times PPV}{SN+PPV},$$

(7) $$MCC=\frac{TP\times TN-FP\times FN}{\sqrt{\left(TP+FN)\times (TN+FP)\times (TP+FP)\times (TN+FN\right)}},$$

where the true positive (TP) is represented as the number of actual PPIs which are predicted correctly by our model; the false negative (FN) is the number of true interacting proteins that are missed; the true negative (TN) is the number of true non-interacting pairs that are predicted correctly, and the false positive (FP) is the number of true non-interacting pairs that are predicted as interacting proteins. In our experiment, the ACC is the proportion of true results (the percentage of correctly identified interacting and noninteracting protein pairs) among the total number of samples. The SN is the proportion of interacting protein pairs that are correctly identified. The Spec measures the proportion of noninteracting protein pairs that are correctly identified. The PPV and NPV are the probability that positive and negative prediction are correct, respectively. The $F_{score}$ is a weighted average of the SN and PPV. It considers both the SN and the PPV of the test to compute the score. The MCC is a more stringent measure of taking into account true and false positives and negatives. Furthermore, it is a correlation coefficient between the observed and predicted binary classifications. The MMC returns a value in [−1,+1]. A coefficient of −1 indicates the disagreement between prediction and real facts, 0 is nearly random prediction, and +1 represents a perfect prediction of PPIs.

### 2.3. Experimental Environment

In this paper, our proposed sequence-based PPIs predictor is implemented using MATLAB (R2009a, the MathWorks, Inc., Natick, MA, USA). All programs are carried out on a computer with 2.5 GHz 6-core CPU, 32 GB memory and Windows operating system(Microsoft Corporation, Redmond, WA, USA). Two RF parameters, the number of decision trees and split are 1000 and 30.

### 2.4. Performance of PPI Prediction

We use eight different PPI datasets to evaluate the performance of our proposed method. The proposed approach is compared with other usual methods on S.cerevisiae, H.pylori and Human datasets. Then, we test our method on five other datasets, including H.sapiens, M.musculus, H.pylori, C.elegans, and E.coli.

#### 2.4.1. Performance on the S.cerevisiae Dataset

We use the first PPI dataset as investigated in You et al. [16] to evaluate the performance of our model.

##### Performance of HOG and SVD

In order to understand the contribution of feature representation components, we test the performance of HOG and SVD for PPI prediction. We use the S.cerevisiae dataset, which is randomly divided into five subsets via a five-fold cross validation. Among them, four subsets are used for training and the remaining set for testing. The cross validation can minimize the impact of data dependency to improve the reliability of experimental results. The prediction result is shown in Table 1. Average accuracies for HOG, SVD and ensemble representation are 93.86%, 92.93% and 94.83%, respectively. Obviously, the HOG approach has better performance than the SVD method. Using ensemble representation, the average accuracy can be raised by 0.97 percentage points.

##### Five-Fold Cross-Validation Results

The prediction result of our method on the S.cerevisiae dataset is shown in Table 2. The average accuracy, precision, sensitivity, and MCC are 94.83%, 97.26%, 92.40%, and 89.77%, respectively. Standard deviations of these criteria values are 0.26%, 0.31%, 0.5%, and 0.50%, respectively. High accuracies and low standard deviations of these criterion values show that our proposed model is effective and stable for predicting PPIs.

##### Comparing with Existing Methods

We compare the prediction performance of our proposed method with that of other existing methods on the S.cerevisiae dataset, as shown in Table 3.

It can be observed that high prediction accuracy of 94.83% is obtained for our proposed model. We use the same S.cerevisiae PPI dataset, and compare our experimental result with You et al. [15,16,23], Wong et al. [19], Guo et al. [11], Zhou et al. [13], Yang et al. [14], where Random Forest (RF), Ensemble Extreme Learning Machines (EELM), Support Vector Machine (SVM), Rotation Forest, Support Vector Machine (SVM), or k-Nearest Neighbor (KNN) is performed with MLD, AC + CT+LD + Moran autocorrelation (MAC), Multi-scale Continuous and Discontinuous (MCD), PR-LPQ, AC, ACC, or LD scheme as the input feature vectors, respectively. Their prediction accuracies are 94.72%±0.43%, 87.00%±0.29%, 91.36%±0.36%, 93.92%±0.36%, 89.33%±2.67%, 87.36%±1.38%, 88.56%±0.33%, and 86.15%±1.17%, respectively, whereas our prediction accuracy is 94.83%±0.26%. Our method has the highest prediction accuracy on the S.cerevisiae PPI dataset, compared with all of the above methods. Our method has the best performance in the Matthew’s correlation coefficient, and the prediction MCC of our method is also the best.

#### 2.4.2. Performance on the H.pylori Dataset

In order to highlight the advantage of our method, we also test it on the H.pylori dataset described by Martin et al. [22]. We compare the prediction performance between our proposed method and other previous works including MLD [15], AC + CT + LD + MAC [23], MCD [16], Discrete Cosine Transformation (DCT) + Substitution Matrix Representation (SMR) [17], LD [13], phylogenetic bootstrap [24], signature products [22], K-local hyperplane distance nearest neighbor algorithm (HKNN) [25], ensemble of HKNN [26] and boosting. In Table 4, we can see that the average prediction performances of our method, such as sensitivity, PPV, accuracy and MCC achieved by proposed predictor, are, 88.15%, 89.79%, 89.06% and 78.15%, respectively. The prediction accuracy of our method is better than all of the above methods, and the prediction PPV of our method is also the best.

#### 2.4.3. Performance on Human Dataset

We also test our method on the Human dataset, which is used by Huang et al. [17]. We compare the prediction performance between our proposed method and Huang’s work [17] on Human dataset, as showed in Table 5. Our method achieves the results that prediction accuracy, sensitivity and MCC are 97.60%, 96.37% and 95.21%, respectively. The prediction accuracy, sensitivity and MCC reported by Huang et al. [17] are 96.30%, 92.63% and 92.82%, respectively. Again, our method obtains better prediction results than Huang’s work on the Human dataset, in terms of accuracy and MCC.

### 2.5. PPI Identification on Independent across Species Datasets

Our test on the two datasets above shows very good prediction results. In addition, our methods are tested on five other independent species’ datasets. If a large number of physically interacting proteins in one organism exist in a co-evolved relationship, their respective orthologs in other organisms interact as well. In this section, we use all 11,188 samples of the S.cerevisiae dataset as the training set and other species datasets (E.coli, C.elegans, H.sapiens, H.pylori and M.musculus) as test sets. We use the same feature extraction method as described above. The performance of these five experiments is summarized in Table 6. The accuracies are 93.18%, 90.28%, 94.58%, 92.03%, and 92.25% on E.coli, C.elegans, H.sapiens, H.pylori and M.musculus datasets, respectively. It shows that the model is capable of predicting PPIs from other species. The prediction result of our method is better than You’s work [15], Huang’s work [17] and Zhou’s work [13], in terms of accuracy.

### 2.6. PPI Network Prediction

**Figure 1:**
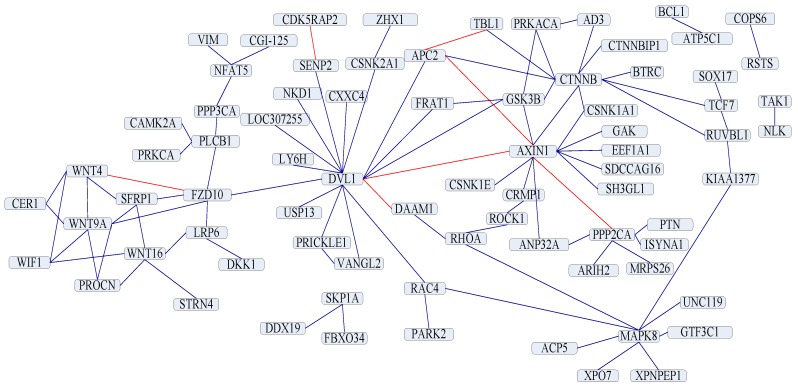
A crossover network for the Wnt-related pathway.

The useful application of the PPI prediction method is the capability of predicting PPI networks. Our method predicts one of the important PPI networks assembled by PPIs pairwise. The Wnt-related network is a typical crossover network, and its related pathway is essential in signal transduction. Ulrich et al. [20] has demonstrated the protein interaction topology of the Wnt-related network. Shen et al. [12] have tested their method on the network. The accuracy of their method is 76.04% in the network: there are 96 PPI pairs in this network, and 73 PPI pairs are predicted correctly by their method. We also try to predict PPIs in the Wnt-related network. The prediction result shows that 89 interactions among 96 PPIs in the network are discovered by our method, and the accuracy is 92.71%, which is better than Shen’s work [12]. The prediction result and the Wnt-related network are shown in Figure 1. Dark blue lines are true prediction, and red lines are false prediction.

### 2.7. Comparison of Different Protein Representation Approaches

Loris Nanni et al. [27,28] described some methods for protein representation matrix containing Amino-Acid Sequence (AAS), Position-Specific Scoring Matrix (PSSM), and Physicochemical Property Response Matrix (PR), and so on. We analyze the performance of BLOSUM62 [18], AAC matrix, AAC + BLOSUM62, AAS, PSSM and PR as protein representation matrix by our method (HOG and SVD algorithm), showed in Table 7. In addition, PR can not use the SVD algorithm, and it is only processed by HOG algorithm. Here, we test these different protein representation matrix on S.cerevisiae, H.pylori and Human datasets, respectively. Accuracy values of AAC matrix by our method are 94.83%, 89.06% and 97.60% on three datasets. Compared to other protein representation methods, the prediction accuracy of AAC is better than all of the above methods on S.cerevisiae and Human datasets.

## 3. Discussion

At present, a lot of computational methods are used to predict PPIs. However, the performance and effectiveness of previous prediction models can still be enhanced. In this paper, we develop a new method for predicting PPIs, via primary sequences of two proteins. The prediction model is constructed based on an ensemble feature representation scheme. We use HOG and SVD to improve the performance in predicting PPIs, via Random Forest. To test the performance of the AAC matrix, we compare it with other common protein representation approaches. These approaches include BLOSUM62, AAS, PSSM and PR, which represent a protein sequence as a matrix. In addition, these representation matrices are extracted feature by HOG and SVD algorithm. The performance of our method is better than all of the above methods on the S.cerevisiae and Human datasets.

From the experimental results, our method is applied to three datasets and the prediction ability of our approach is better than that of other existing state-of-the-art PPI prediction methods. The prediction result shows that our method achieves 94.83% accuracy on the S.cerevisiae dataset. Our method achieves 89.06% accuracy for the H.pylori PPI dataset. On the Human dataset, the experimental results show that our method achieves 97.60% accuracy. In addition, our proposed method has also obtained good prediction accuracy on cross-species experiments of five other independent datasets. In addition, the proposed method achieves more than 90% accuracy on E.coli, C.elegans, H.sapiens, H.pylori and M.musculus datasets, respectively. Our results indicate that the proposed model can be successfully applied to other species, where experimental PPI data is not available. It should be noticed that the biological hypothesis of mapping PPIs from one species to another species is that large numbers of physically interacting proteins in one organism are co-evolved.

The most important issue of PPI prediction methods is the accurately predicting PPI networks. We extend our method to predict an important PPI network, and the accuracy of our method is increased 16.67% compared with CT. General PPI networks are crossover networks, so our method is useful in practical applications. All of these results verify that our proposed method is a very useful support tool for future PPI network research. Because the proposed method adopts an effective feature extraction method and captures useful protein sequence information, the performance of our method is good on above data sets. In future work, we will extend our method to predict other important PPI networks.

## 4. Materials and Methods

In this paper, we propose a novel method to extract features from protein sequences, for predicting protein–protein interactions. First, we construct Amino Acid Contact (AAC) matrix, based on 6323 protein–protein complexes from the Protein Data Bank. We use an AAC matrix to represent the protein sequence as a Substitution Matrix Representation (SMR) matrix. Then, we use Histogram of Oriented Gradient (HOG) and Singular Value Decomposition (SVD) algorithms to extract the feature vector from the SMR matrix. Finally, we feed the feature vector into a specific classifier for PPI prediction.

### 4.1. Amino Acid Contact Matrix

Inspired by previous work [29], we consider 20 amino acid types and one solvent contacting residues in protein surfaces. The Amino Acid Contact (AAC) matrix is obtained from the statistical analysis of residue-pairing frequencies in one protein–protein complex database. We select 6323 complexes from the Protein Data Bank [30]. These complexes are made up of two or more protein subunits and their structures are determined by X-rays with cutoff values of resolution 2.2 Å and sequence identity 30%. We define a pair of residues from two subunits as a contact pair, if two atoms (one from each subunit) are within distance *d* (set to be 6 in our method).

The AAC matrix is correlated to statistical observed numbers of pairwise contacts on the interface. The amino acid contact between two amino acid types *i* and *j* is defined as follows:

(8) $$AAC\left(i,j\right)=-\ln\frac{N_{i,j}/C_{i,j}}{\left(N_{i,0}/C_{i,0}\right)\times \left(N_{j,0}/C_{j,0}\right)},$$

where type 0 corresponds to the solvent. The number of *i*-*j* contact is defined as $N_{i,j}=\sum_{p}n_{ij,p}$, and the number of *i*-0 contact is defined as $N_{i,0}=\sum_{p}n_{i0,p}$. These values are the estimation of actual numbers of contacts, where $n_{ij,p}$ is the contact number between residue types *i* and *j*, and $n_{i0,p}$ is the contact number between residue type *i* and water in each complex. In addition, the expected number of contacts is defined as follows:

(9) $$C_{i,j}=\sum_{p}n_{rr,p}\times \frac{n_{i,p}n_{j,p}}{n_{r,p}^{2}},$$

and

(10) $$C_{i,0}=\sum_{p}n_{r0,p}\times \frac{n_{i,p}}{n_{r,p}},$$

where *p* denotes a complex of protein pair in the data set; $n_{i,p}/n_{r,p}$ is the fraction of residue type *i* in all residues for each complex; $n_{rr,p}$ and $n_{r0,p}$ are total numbers of residue–residue contacts and residue–water contacts in each complex, respectively.

### 4.2. Substitution Matrix Representation

We represent the protein sequence as a Substitution Matrix Representation (SMR) matrix, mentioned by Yu et al. [31] and Huang et al. [17]. The given *L*-length protein sequence can be represented as one 20×L matrix, based on a substitution matrix. We use the above AAC matrix as the substitution matrix, which is used for replacing a residue–water contact with a residue–residue contact. SMR(i,j) represents the distance of *i*-type of amino acid contacting to *j*-position of the given protein sequence in the interaction process, which is defined as follows:

(11) $$SMR\left(i,j\right)=AAC\left(i,p_{j}\right),$$

where i=1,…,20 is one of twenty amino acid types, j=1,…,L is one of *L* positions in the given protein sequence, and $p_{j}$ is the amino acid type of *j*-position. AAC denotes the 20×20 substitution matrix.

### 4.3. Histogram of Oriented Gradient

In Nanni’s work [32], they explored a method for representing a protein as an image and extracted features from the image using continuous wavelet transform for protein classification. In this paper, the Histogram of Oriented Gradients (HOG) [33,34] is a feature descriptor, used in computer vision and image processing for the purpose of object detection. In our work, SMR can be regarded as a special images matrix, which contains the AAC information.

**Figure 2:**
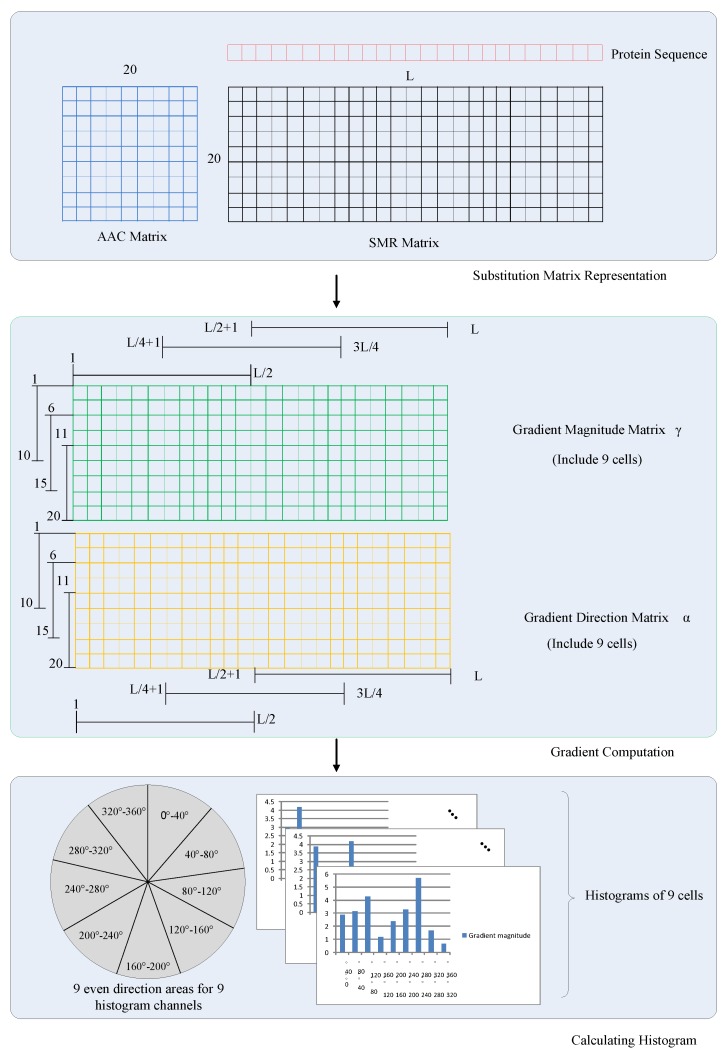
The schematic diagram for calculating Histogram of Oriented Gradient (HOG).

The essential thought of applying the HOG descriptor is that local object appearance and shape can be described by the distribution of intensity gradients, which can be used to describe local detail features of the signal, and the schematic diagram of HOG is shown in Figure 2.

#### 4.3.1. Gradient Computation

The most common method of gradient computation is to apply the one-dimensional centered point discrete derivative mask in both of the horizontal and vertical directions. Gradient values $G_{horizontal}\left(i,j\right)$ and $G_{vertical}\left(i,j\right)$ represent the horizontal and vertical directions, which can be computed as follows:

(12) $$G_{horizontal}\left(i,j\right)=\left\{\begin{array}{ll} SMR(i+1,j)-0, & i=1,\\ SMR(i+1,j)-SMR(i-1,j), & 1<i<20,\\ 0-SMR(i-1,j), & i=20, \end{array}\right.$$

(13) $$G_{vertical}\left(i,j\right)=\left\{\begin{array}{ll} SMR(i,j+1)-0, & j=1,\\ SMR(i,j+1)-SMR(i,j-1), & 1<j<L,\\ 0-SMR(i,j-1), & j=L. \end{array}\right.$$

Then, the gradient magnitude γ(i,j) and the gradient direction α(i,j) can be calculated as follows:

(14) $$\gamma \left(i,j\right)=\sqrt{G_{horizontal}{\left(i,j\right)}^{2}+G_{vertical}{\left(i,j\right)}^{2}},$$

(15) $$\alpha \left(i,j\right)=tan^{-1}\left(\frac{G_{vertical}\left(i,j\right)}{G_{horizontal}\left(i,j\right)}\right).$$

Here, we get the gradient magnitude matrix *γ* and the gradient direction matrix *α*, which are two 20×L matrices. The gradient magnitude of *γ* matrix are corresponding to the *α* matrix. Values of the gradient direction is evenly spread over 0 to 360 degrees.

#### 4.3.2. Dividing Matrix and Calculating Histogram

The gradient magnitude matrix *γ* and the gradient direction matrix *α* can be divided into 9 sub-matrices with the same size. Each cell within one sub-matrix contains information of the gradient magnitude and the gradient direction. There are overlapping edge region between each cell to simplify the calculation and divide region. As a result, the information is continuous between each sub-matrix. The location relational mapping between sub-matrix and matrix is defined as follows:

(16) $$\gamma_{p,q}\left(a,b\right)=\gamma \left(5\times p+1+a,q\times \frac{L}{4}+1+b\right),$$

(17) $$\alpha_{p,q}\left(a,b\right)=\alpha \left(5\times p+1+a,q\times \frac{L}{4}+1+b\right),$$

where *p* and *q* are subscripts of the sub-matrix (0≤p≤2, 0≤q≤2, the total is 9), and *a* and *b* are inside location subscripts of the sub-matrix (0≤a≤9, $0\leq b\leq \frac{L}{2}-1$).

For every sub-matrix, we create 9 orientation-based histogram channels on account of the gradient direction, including 0${}^{\circ }$–40${}^{\circ }$, 40${}^{\circ }$–80${}^{\circ }$, …, 320${}^{\circ }$–360${}^{\circ }$. Then, we cast the weighted vote for each orientation-based histogram channel, based on the gradient magnitude. In the sub-matrix *k* (k=3×p+q+1), the gradient direction $\alpha_{p,q}\left(a,b\right)$ determines the histogram channel ch to which the cell belongs, and the corresponding histogram channel $v_{k}\left(ch\right)$ is increased by the gradient magnitude $\gamma_{p,q}\left(a,b\right)$.

Since for each sub-matrix we can get 9 histogram channels, we will obtain 9×9=81 channels for 9 sub-matrices. Therefore, we get a vector $v=(v_{1}\left(1\right),v_{1}\left(2\right),\ldots ,v_{1}\left(9\right),v_{2}\left(1\right),\ldots ,v_{9}\left(9\right))$ from one protein sequence.

#### 4.3.3. Normalization

To obtain the invariance in every local matrix, we normalize the vector *v*. The normalization factor $f_{HOG}$ can be calculated as follows:

(18) $$f_{HOG}=\frac{v}{\sqrt{∥v∥+\epsilon }},$$

where *ϵ* is a small constant, and here we set it as 0.01.

### 4.4. Singular Value Decomposition

In linear algebra, the Singular Value Decomposition (SVD) is a factorization of a real or complex matrix. The SVD is often used for image signal compression and de-noising. Formally, SVD of one m×n matrix *M* is a factorization of the form as follows:

(19) $$M=U\Sigma V^{*},$$

where *U* is a real or complex unitary matrix (m×m), Σ is a rectangular diagonal matrix with nonnegative real numbers on the diagonal (m×n), and $V^{*}$ is a real or complex unitary matrix (n×n). The diagonal entries of Σ are known as the singular values of *M*. The columns of *U* and the columns of *V* are called left-singular vectors and right-singular vectors of *M*, respectively.

We apply SVD to decompose the transposed matrix of the SMR matrix $SMR^{T}$, in order to extract fixed-size features from variable-length protein sequences. SVD could acquire the potential pattern of the original matrix, and $V^{*}$ can get 20×20 entries. Therefore, we get a vector $f_{SVD}$ by all entries $\left(V_{1,1}^{*},V_{1,2}^{*},\ldots ,V_{1,20}^{*},V_{2,1}^{*},\ldots ,V_{20,20}^{*}\right)$.

### 4.5. Random Forest Classifier

In this paper, the feature space of each pair of proteins is composed of HOG and SVD. Specifically, we extract 81+400=481 features to be encoded to represent one protein sequence. Therefore, each pair of proteins can be encoded to be represented as 481×2=962 features $F=(f_{HOG},f_{SVD})$. We define 962-dimentional feature vector $F=(f_{1},f_{2},\ldots ,f_{962})$ as the input data of the classifier model. The class label *t* of interacting pair or non-interacting pair is set as 1 or −1, respectively.

We feed the feature vector into a Random Forest model for judging interaction pairs and non-interaction pairs. Random Forest (RF) is an algorithm for classification developed by Leo Breiman [35], which uses an ensemble of classification trees. Each classification tree is built by using a bootstrap sample of the training data, while each split candidate set is a random subset of variables. The bagging and random variable selection can cause low correlation of individual trees. RF has been demonstrated to have excellent performance in classification tasks.

We randomly choose *N* cases from the original data with replacement for building the training set to grow the classification tree. At each node, *k* variables are selected at random out of *K* input variables (k<<K and K=962), and the best split on these *k* variables is used to split the node. The value of *k* is held constant during the forest growing. For new cases, classification results can be obtained by the voting method on these trees.

## 5. Conclusions

In this paper, we develop a new method for predicting PPIs by primary sequences of two proteins. The prediction model is constructed based on random forest and an ensemble feature representation scheme (HOG and SVD feature). From the experimental results, it can be seen that the prediction performance of the proposed method is better than that of previous methods on several common data sets. What’s more, we extend our method to predict an important PPI network, and the accuracy of our method is obviously higher than that of the CT. All these results demonstrate that our proposed method is a very promising and useful support tool for future proteomics research.

## Figures and Tables

**Table 1:** Analyze the performance of the Histogram of Oriented Gradient (HOG) and Singular Value Decomposition (SVD) on S.cerevisiae dataset by Random Forest (RF) classifier.

Feature	Classifier	ACC (%)	SN (%)	Spec (%)	PPV (%)	NPV (%)	F1 (%)	MCC (%)
HOG	RF	93.86 ± 0.47	90.67 ± 0.47	97.05 ± 0.74	96.86±0.72	91.22 ± 0.64	93.66 ± 0.40	87.90 ± 0.94
SVD	RF	92.93 ± 0.52	90.25 ± 0.70	95.59 ± 1.36	95.38 ± 1.22	90.76 ± 0.42	92.74 ± 0.47	85.99 ± 1.10
HOG + SVD	RF	94.83 ± 0.26	92.40 ± 0.50	97.26 ± 0.31	97.10 ± 0.35	92.79 ± 0.59	94.69 ± 0.24	89.77 ± 0.50

**Table 2:** Five-fold cross validation result obtained by using our proposed method on the S.cerevisiae dataset.

Testing Set	ACC (%)	SN (%)	Spec (%)	PPV (%)	NPV (%)	F1 (%)	MCC (%)
1	94.73	92.70	96.72	96.53	93.08	94.58	89.52
2	95.13	92.80	97.31	97.01	93.51	94.86	90.31
3	95.04	92.67	97.47	97.40	92.84	94.98	90.19
4	94.81	92.24	97.40	97.27	92.59	94.69	89.75
5	94.46	91.60	97.39	97.28	91.91	94.35	89.09
Average	94.83 ± 0.26	92.40 ± 0.50	97.26 ± 0.31	97.10 ± 0.35	92.79 ± 0.59	94.69 ± 0.24	89.77 ± 0.50

**Table 3:** Comparison of the prediction performance between our proposed method and other state-of-the-art works on the S.cerevisiae dataset. N/A means not available.

Method	Feature	Classifier	ACC (%)	SN (%)	PPV (%)	MCC (%)
Our method	HOG + SVD	RF	94.83 ± 0.26	92.40 ± 0.50	97.10 ± 0.35	89.77 ± 0.50
You’s work [15]	MLD	RF	94.72 ± 0.43	94.34 ± 0.49	98.91 ± 0.33	85.99 ± 0.89
You’s work [23]	AC + CT + LD + MAC	E-ELM	87.00 ± 0.29	86.15 ± 0.43	87.59 ± 0.32	77.36 ± 0.44
You’s work [16]	MCD	SVM	91.36 ± 0.36	90.67 ± 0.69	91.94 ± 0.62	84.21 ± 0.59
Wong’s work [19]	PR-LPQ	Rotation Forest	93.92 ± 0.36	91.10 ± 0.31	96.45 ± 0.45	88.56 ± 0.63
Guo’s work [11]	ACC	SVM	89.33 ± 2.67	89.93 ± 3.68	88.87 ± 6.16	N/A
Guo’s work [11]	AC	SVM	87.36 ± 1.38	87.30 ± 4.68	87.82 ± 4.33	N/A
Zhou’s work [13]	LD	SVM	88.56 ± 0.33	87.37 ± 0.22	89.50 ± 0.60	77.15 ± 0.68
Yang’s work [14]	LD	KNN	86.15 ± 1.17	81.03 ± 1.74	90.24 ± 1.34	N/A

* The feature representation of protein-protein interaction include the Histogram of Oriented Gradient (HOG), Singular Value Decomposition (SVD), Multi-scale Local Descriptor (MLD), Auto-Correlation (AC), Conjoint Triads (CT), Local Descriptors (LD), Moran autocorrelation (MAC), Multi-scale Continuous and Discontinuous (MCD), Local Phase Quantization descriptor (PR-LPQ) and Auto Cross Covariance (ACC). The classifiers include the Random Forest (RF), Ensemble Extreme Learning Machine (E-ELM), Support Vector Machine (SVM) and K-Nearest Neighbor (KNN).

**Table 4:** Comparison of the prediction performance between our proposed method and other methods on the H.pylori dataset. N/A means not available.

Methods	ACC (%)	SN (%)	PPV (%)	MCC (%)
Our method	89.06	88.15	89.79	78.15
You’s work (MLD) [15]	88.30	92.47	85.99	79.19
You’s work (AC + CT + LD + MAC) [23]	87.50	88.95	86.15	78.13
You’s work (MCD) [16]	84.91	83.24	86.12	74.40
Huang’s work (DCT + SMR) [17]	86.74	86.43	87.01	76.99
Zhou’s work [13]	84.20	85.10	83.30	N/A
Phylogenetic bootstrap [24]	75.80	69.80	80.20	N/A
HKNN [25]	84.00	86.00	84.00	N/A
Signature products [22]	83.40	79.90	85.70	N/A
Ensemble of HKNN [26]	86.60	86.70	85.00	N/A
Boosting	79.52	80.37	81.69	70.64

**Table 5:** Comparison of the prediction performance between our proposed method and other methods on the Human dataset.

Methods	ACC (%)	SN (%)	PPV (%)	MCC (%)
Our method	97.60	96.37	98.59	95.21
Huang’s work (DCT + SMR) [17]	96.30	92.63	99.59	92.82

**Table 6:** Prediction results on five independent species by our proposed method, based on the S.cerevisiae dataset as the training set. N/A means not available.

Species	Testing Pairs	ACC(%)			
		Our Method	You’s Work [15]	Huang’s Work [17]	Zhou’s Work [13]
E.coli	6954	93.18	89.30	66.08	71.24
C.elegans	4013	90.28	87.71	81.19	75.73
H.sapiens	1412	94.58	94.19	82.22	76.27
H.pylori	1420	92.03	90.99	82.18	N/A
M.musculus	313	92.25	91.96	79.87	76.68

**Table 7:** Comparison of different protein representation approaches by our method.

Dataset	ACC(%)					
	AAC	BLOSUM62	AAC + BLOSUM62	PSSM	AAS	PR
S.cerevisiae	94.83 ± 0.26	94.32 ± 0.21	94.34 ± 0.63	94.21 ± 0.57	94.19 ± 0.66	93.37 ± 0.38
H.pylori	89.06 ± 0.96	88.62 ± 1.13	89.16 ± 1.09	88.51 ± 1.04	87.59 ± 1.27	84.67 ± 1.29
Human	97.60 ± 0.29	97.56 ± 0.13	97.59 ± 0.16	97.55 ± 0.33	97.46 ± 0.48	96.56 ± 0.91
